# Bioactive Ceramic Scaffolds for Bone Tissue Engineering by Powder Bed Selective Laser Processing: A Review

**DOI:** 10.3390/ma14185338

**Published:** 2021-09-16

**Authors:** Nikhil Kamboj, Antonia Ressler, Irina Hussainova

**Affiliations:** 1 Department of Mechanical and Industrial Engineering, Tallinn University of Technology, Ehitajate Tee 5, 19086 Tallinn, Estonia; nikhil.kamboj@taltech.ee; 2 Faculty of Chemical Engineering and Technology, University of Zagreb, Marulićev Trg 19, p.p.177, HR-10001 Zagreb, Croatia; aressler@fkit.hr

**Keywords:** additive manufacturing, bone tissue engineering, ceramics, calcium phosphate, calcium silicate, critical-sized defects, drugs, laser powder bed fusion, scaffolds, selective laser melting, selective laser sintering

## Abstract

The implementation of a powder bed selective laser processing (PBSLP) technique for bioactive ceramics, including selective laser sintering and melting (SLM/SLS), a laser powder bed fusion (L-PBF) approach is far more challenging when compared to its metallic and polymeric counterparts for the fabrication of biomedical materials. Direct PBSLP can offer binder-free fabrication of bioactive scaffolds without involving postprocessing techniques. This review explicitly focuses on the PBSLP technique for bioactive ceramics and encompasses a detailed overview of the PBSLP process and the general requirements and properties of the bioactive scaffolds for bone tissue growth. The bioactive ceramics enclosing calcium phosphate (CaP) and calcium silicates (CS) and their respective composite scaffolds processed through PBSLP are also extensively discussed. This review paper also categorizes the bone regeneration strategies of the bioactive scaffolds processed through PBSLP with the various modes of functionalization through the incorporation of drugs, stem cells, and growth factors to ameliorate critical-sized bone defects based on the fracture site length for personalized medicine.

## 1. Introduction

**Figure 1:**
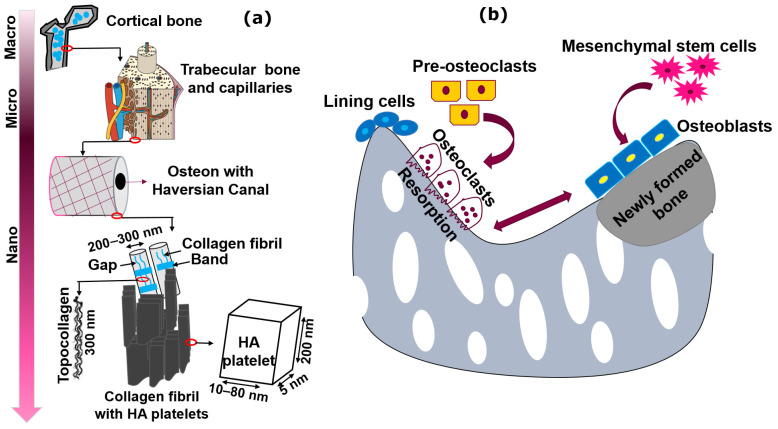
(**a**) Bone hierarchy at macro-, micro- and nanolevel, and (**b**) bone regeneration process involving osteoblasts and osteoclasts.

Osseous tissue plays a pivotal role in the body, serving several functions such as locomotion, support, protection of internal organs, production of blood cells and storage of ions, etc. Cell performance is a critical parameter for many physiological and pathological processes, as well as for anabolic and catabolic activities. Figure 1 demonstrates the bone hierarchy at macro-, micro-, and nanolevel with the schematic representation of the bone regeneration process involving osteoblasts and osteoclasts.

The worldwide incidence of bone diseases and disorders requiring arthroplasty and/or other surgical intervention is persistently increasing. A particular challenge in ongoing developments for the treatments of orthopedic critical injuries originates from the need to balance design criteria and material biocompatibility with the mechanical and geometric constraints regulated by the physiological environment of the bone defect. Appropriate material designs for bone tissue engineering (BTE) require a deep understanding of the architecture, hierarchy, and composition of native bone tissue and the ability to mimic its physiochemical properties.

The bone is a natural composite of collagen type I (COL-1) and nanosized calcium-deficient carbonated hydroxyapatite crystals deposited in parallel with the collagen fibers. The hard, bony tissue at the nanolevel is a combination of strength and ductility [1], which enables energy to be absorbed prior to bone fracture. At the microscale level, an osteon is a basic unit for cortical bone. The dense and trabecular bone both comprise collagen fibrils [2], which are reinforced with hydroxyapatite (HA) particles as depicted in Figure 1. At the macrolevel, the bone can be contemplated as a composite of osteons and body fluids. Mimicking the features of natural tissue is a nontrivial task requiring special fabrication methods and techniques, as well as mechanically reliable and biophysically compatible materials. Fulfilling the intricate design of bioinspired scaffolds is hardly possible with traditional manufacturing methods due to the complex architecture of bone. The conventional techniques (e.g., freeze-drying, solvent casting, electrospinning, sol−gel, gas foaming, etc.) have several drawbacks in the forming and fabricating of complex shapes, such as the low mechanical properties of produced coupons, the use of toxic organic solvents, large energy utilization, the evolution of additional phases in synthetic matrices, etc. Recent progress in additive manufacturing (AM) has provided a platform to produce biomimetic components with geometric freedom [3] and the possibility of in situ development of functional structures.

Biomaterials can be described as a natural or synthetic supporting structure, which consequently can be inserted into body tissue in the form of a medical device or as synthetic grafts. Metallic scaffolds are confronted with numerous shortcomings, such as restrained degradation and stress-shielding phenomenon, which may have severe consequences on the regrowth of osseous tissue at the site of injury or implantation [4]. Synthetic polymeric scaffolds show a foreign body reaction coupled with immunological reactions when implanted at the affected sites, which can be attributed to the lixiviation of toxic microparticles and deteriorating immunomodulation effects [4,5]. Bioactive ceramics are frequently used biomaterials in BTE as the supporting structure at the site of injury and are in the ascendency when compared to bioinert ceramics, not only because of their ability to directly bond with surrounding bone tissue but they can also be used as synthetic grafts for open and/or long bone critical defects. Often CaP, but mainly HA scaffolds are effectively used as they simulate or replicate the natural inorganic part of the bone tissue. Recently, CS and doped CS with different trace elements (Mg, Zn, and Zr) have been widely used in BTE for their superior mechanical properties [6]. Therefore, this review article will mostly focus on the synthetic bioactive ceramics encompassing CaP, CS, and their composites.

**Figure 2:**
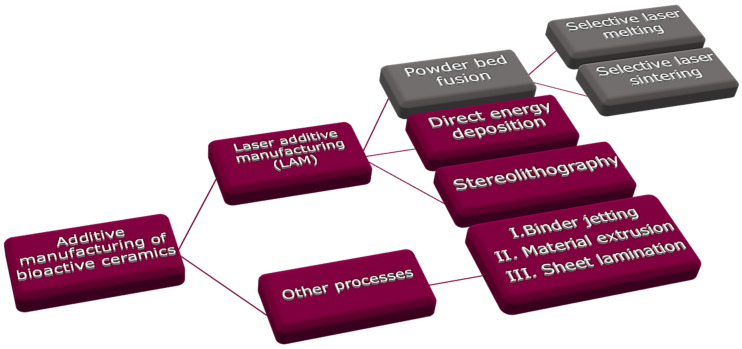
AM techniques used for bioactive ceramics. The categories labelled in grey are comprehensively reviewed in this study.

The specific target of scaffold functionality in adapting peculiar designs and utilizing novel ceramic materials is diminishing orthopedic ailments, such as donor-site morbidity, bone tumor, non-unions, and clinical constraints. Numerous AM methods have recently been utilized to produce the tailored structures, i.e., L-PBF, material extrusion (ME), stereolithography (SLA), directed energy deposition (DED), binder jetting (3DP), vat polymerization, etc. [7]. Figure 2 illustrates various AM methods for bioactive ceramic processing.

Laser additive manufacturing (LAM) through the powder bed or L-PBF involves two techniques, i.e., (SLS and SLM) or accumulatively called PBSLP. Nowadays, PBSLP has been used as an attractive technique enabling different modes of functionalization of biomaterials through novel engineering to develop drug-delivery systems (DDS) and personalized medicine [8]. PBSLP has the unique potential to directly incorporate active biomolecules, such as drugs and other moieties, directly into the powder bed even though they are temperature sensitive or can be incorporated after the scaffold fabrication [9] and their release can be tailored by tampering with the SLM or SLS parameters [10]. PBSLP has the significant potential to print biomaterials (printlets) with several tailored engineering properties such as immediate, control, multilayered, multireservoir, and visually impaired printlets for targeting specific patient groups for personalized medicine [8].

This review’s specific topic is the LAM of bioactive ceramic scaffolds processed only through direct PBSLP using SLM or SLS as shown in Figure 2. The second section provides detailed information on the PBSLP and its advantages over the other AM techniques used for the fabrication of bioactive ceramics. The third section will brief about the biological, architectural, and mechanical requirements of the bioactive ceramic scaffolds. The fourth section presents bioactive ceramic scaffolds based on CaP and CS and their respective composites processed through a direct L-PBF approach (SLS/SLM). In the last section, an effort has also been made to convey the practical aspects of PBSLP for the bioactive scaffolds (CaP and CS) as a potential technique for the various modes of functionalization through the incorporation of drugs, stem cells, and growth factors to ameliorate critical-sized bone defects based on the fracture site length for a specific clinical target. The potential developments for fabrication through PBSLP and the design of bioactive ceramics are also discussed.

## 2. Powder Bed Fusion of Ceramics

**Figure 3:**
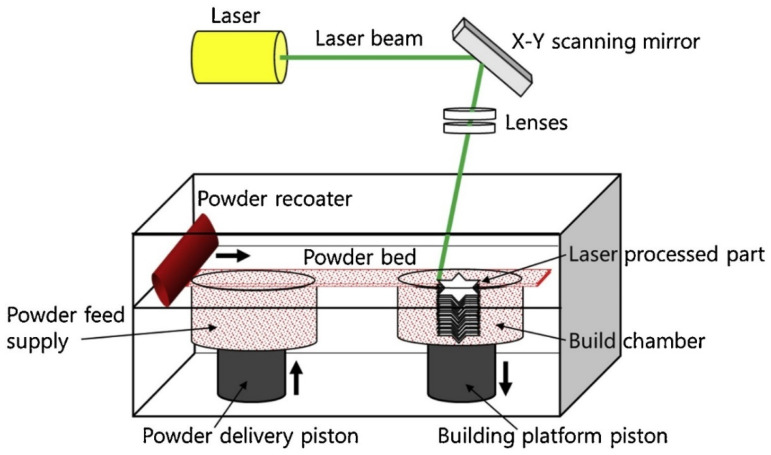
Schematic of powder bed fusion process, in accordance to Pfeiffer et al., adapted from [12].

L-PBF through SLM and SLS, is an AM technique allowing complex geometries to be built directly from three-dimensional (3D) computer-aided design (CAD) models layer by layer with a high-intensity laser beam. The powder particles are selectively fused or sintered together with a laser beam in a layer-wise manner, as demonstrated in Figure 3. The binding mechanism unfolding during SLM and SLS processes can be broadly classified under four categories, which include solid-state sintering of the ceramic particles, liquid-phase sintering (indirect powder bed fusion of ceramic particles involving binders), partial melting (direct powder bed fusion of the ceramic particles without binders), and melting [11]. Recently, SLS has been extensively studied for polymers, metals, and their composites and demonstrated excellence in the direct development of products with intricate shapes.

However, the process of manufacturing crack-free dense ceramic coupons is rather difficult due to their high melting point, poor thermal shock resistance, and low or no plasticity. Moreover, sintered parts often exhibit poor mechanical properties, which may be attributed to the rapid heating and cooling in the powder bed, which result in cracking, unwanted porosity and therefore, renders them unsuitable for particles diffusion [13].

**Figure 4:**
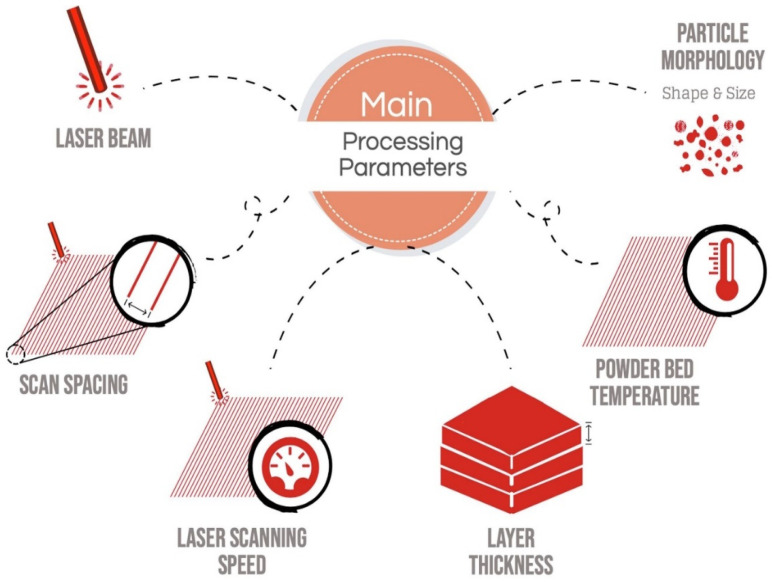
Illustration of the process parameters involved in PBSLP, adapted with permission from [8].

The ultimate performance of the sintered bioactive ceramic product greatly depends on the processing parameters of PBSLP outlined in Figure 4, which must be carefully optimized in order to achieve the intended structures for a specific application. In this review, we will only be discussing direct PBSLP of the bioactive ceramic scaffolds, since no review article in the literature has been entirely dedicated to the direct PBSLP of the bioactive ceramic scaffolds. Indirect PBSLP involves specific binders and laborious steps such as debinding and firing (degrades the chemical properties of the fabricated part) and additional post-treatment processing (e.g., sintering), which can ultimately deteriorate the functionality of the fabricated constructs [12]. The main process parameters to be considered are laser-beam type, laser current, scan spacing, orientation, laser scanning speed (which in turn depends on point distance and exposure time), scan spacing and layer thickness. The laser power and laser scanning speed are interrelated parameters since they determine the amount of energy transferred to the powder bed of a certain area. Modulating the laser scanning speed has a profound effect on the fabricated construct. A high laser scanning speed may consequently lead to inadequate sintering of the particles or partial melting, while a low scanning speed may eventually lead to the excessive evaporation of the particles in the bioactive powder bed, which affects the formation of the pores and imperfections resulting ultimately in the degradation of the product’s functionality [14].

The hatching distance or scan spacing, or line offset is regarded as the distance between the adjoining laser beam tracks. The scan spacing in PBSLP can affect the quality of the product, since it is obligatory to find the appropriate distance between the two neighboring tracks to avert the zones with no adequate interaction with laser and powder bed. This can have two consequences: either unreacted powder or excessive evaporation of the material [8].

In the PBSLP, it is feasible to change the laser spot diameter, which can have a profound influence on the laser scanning speed, by focusing or defocusing the laser spot size [15]. This would have reflective characteristics on the amount of energy transferred to the powder bed. The amount of energy transferred to the powder bed or energy density is calculated using Equation (1) [16],

(1) E=Peff/(Vs∗hd∗d)

where E is the laser energy density, Peff is the laser power, Vs is the laser scanning speed, hd is the hatch offset and d is the layer thickness.

According to Equation (1), a lower scanning speed increases the energy density and, therefore, increases the contact time between the laser and powder bed. The energy density window has to be carefully designed to achieve the desired functional scaffold for BTE [17].

The PBSLP used for the sintering/melting of the ceramic powder bed is usually operated with the fiber Nd:YAG laser with a wavelength of λ = 1.064 μm or a CO_2_ laser which is often used in the medical industry to print large and small prototypes with a wavelength of λ = 10.64 μm and power between 40 to 200W. The CO_2_ laser can be substituted with a CO laser for the fabrication of medical implants of a high printing precision [8]. However, the fiber laser can achieve the finest resolution for ceramic materials for the fabrication of contorted shapes [18] as the fine spot size posed by the fiber laser gives several advantages, such as the large transmission of energy to the powder-bed and scale-down time needed to sinter or melt the powder [19].

Generally, the morphology and size of the powder particles for laser sintering are required to be of a specified scale and spherical shape providing good flowability and packing density, and, consequently, under-sintered areas and voids are almost unavoidable. Large particles in the PBSLP ranging between 200 and 400 μm require more energy transfer from the laser [20,21]. Nanosized particles tend to agglomerate due to an electrostatic interaction between them. In order to revamp the powder flow characteristics, it is recommended [22,23] that a multimodal powder feedstock with a bimodal size distribution is used, since the fine particles can fill the interstitial gaps and maximize the densification of the fabricated part. Additionally, the distribution of the particle size should be as narrow as possible to ensure homogeneous energy absorption in the powder bed.

The scanning strategy (zig-zag, island, in-out, and out-in scanning) along with printing orientation (diagonal, vertical, and horizontal) also influences the mechanical and physical characteristics of the final product [24,25]. Scanning strategies have consequential effects on the mechanical properties since the heating and cooling rates in the PBSLP can significantly affect tensile properties, ductility, distortion, hardness, and fatigue behavior [26]. Different scanning strategies could result in different microstructures and density, which can be attributed to the differential temperature gradient, the effect of the laser path, and the length of the scan vectors in PBSLP [27]. Additionally, the scanning rotation angles between the successive layers of the different scanning strategies could also influence the densification and, hence, significantly alter the mechanical properties such as the toughness, ductility, and ultimate tensile strength [28].

### 2.1. Advantages of PBSLP Techniques over Other AM Techniques for Bioactive Ceramics

Bioactive bone mimicking has been accomplished via several AM methods as shown in Figure 2. For instance, 3DP was applied to the fabrication of CaP scaffolds with tailored porosity and structural characteristics [29]. However, on the other hand, PBSLP offers several advantages for the fabrication of bioactive scaffolds for BTE when compared not only to the conventional techniques but also to the other AM techniques like ME, 3DP, DED, and SLA. Most of the work has been dedicated to either pure metallic and polymer scaffolds or their respective composites through PBSLP to develop structures maintaining the overall properties of bone. The multiple advantages offered by PBSLP for bioactive scaffolds when compared to other AM techniques are:

(1) Capability of fabrication of drug delivery and healthcare products;
(2) Customized production of the implants;
(3) Cost-effectiveness for the small prototypes;
(4) Various options for designers to formulate complex shapes for fabrication;
(5) Absence of postprocessing stages;
(6) Tailorable porosity of a wider window (100–1000 µm pore sizes), which are generally required in BTE as compared to the other AM techniques used for BTE.

**Figure 5:**
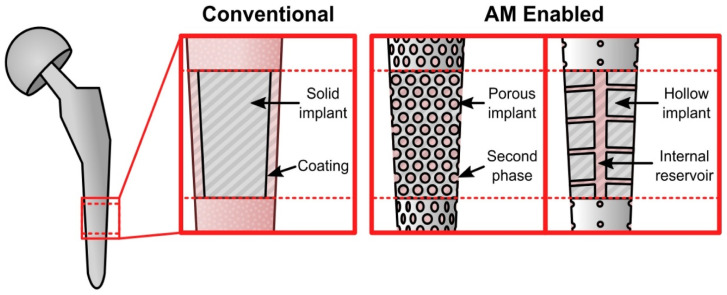
Major differences in the approaches of the conventional technique and PBSLP with the conventional technique depicting the addition of therapeutic coating. AM illustrating two different types of approaches with intercalation of drug into PBSLP-produced bioactive ceramic/metal lattice, and encompassing internal reservoir, adapted with permission from [13].

PBSLP offers an array of advantages when it comes to health care applications [30] allowing the fine-tuning of drug release, the delivery of which is either accelerated or decelerated by changing the laser scanning speed and position of the microchannels in the implant [31]. SLS has the potential to fabricate different unusual designed shapes to customize the needs of the patient with visual impairment [32]. This efficacious approach allows patients to differentiate the medicines based on tactile perception and has proved advantageous when medicines are taken out from the packaging [14]. PBSLP also has the capability to surpass the shortcomings of conventional techniques by incorporating two drugs simultaneously (acting as a multireservoir) [33]. Hence, following the route of the PBSLP process, therapeutic agents or drug vehicle release can be tailored by adjusting the implant pore size and porosity level [34], as depicted in Figure 5. For example, orthopedic bioactive ceramic composite implants assimilating vancomycin were fabricated through SLM for treating periprosthetic infections [35].

PBSLP is peculiarly advantageous in the medical sector where a low volume of personalized items are a prerequisite [36], such as ceramic porous bone implants [37]. In addition, PBSLP allows the building of the final product on demand in a short time span, which consequently reduces the logistics and storage cost. As a result, it drastically decreases the market cost and has promoted the growth and explosion of PBSLP technologies [38].

Direct material fusion, which happens either by sintering (SLS) or by melting (SLM) in the L-PBF is usually a single-step (direct AM) process. Debinding and sintering are peculiar to multistep AM (indirect) technologies like ME, 3DP, and SLA, which typically involve organic binders. Removal of the binders in the debinding step is generally time-consuming and can occasionally lead to the formation of additional bioactive phases [39]. Delamination between the layers and cracking in the scaffolds are the major problems associated with the usage of organic binders in the multistep AM process. The possibility of avoiding the additional bioactive phase formation, along with a crack-free bioactive ceramic scaffold of copper-doped HA, has been demonstrated though powder bed sintering [40].

The lasers can fine-tune the grains of HA bioceramics to obtain a wide range of mechanical properties by modulating the energy density of the CO_2_ laser [41]. It is also possible to temper the grain size of nano-HA from acicular to a near spherical or ellipsoidal shape to achieve partial or full melting [42]. The potential of SLM technology to fabricate CS/silicon bioactive ceramic composites with high compressive strength is unleashed [17,35], It is also possible to achieve higher hardness and good fracture toughness when HA is reinforced into the metal matrix [43,44]. Hence, SLM provides the freedom to interfere with the morphology of the powder because of the rapid heating and cooling process unfolding (solidification at a rate of 105–108 K/s) and to have an upper hand in the other AM processes with regards to superior mechanical properties.

The absence of the melting process in the SLS process (CO_2_ laser) can have several repercussions on the final product. The powder bed selectively scanned by the laser for coalescing the particles significantly reduces the surface energy, and hence causes sintering [45]. The sintering of the particles with the neck formation results in the formation of voids (microspaces) between the two grains [46]. These microspaces play a crucial role in the scaffold for biomimicking the extracellular matrix (ECM) usually found in bone and hence can be used for DDS [35].

**Figure 6:**
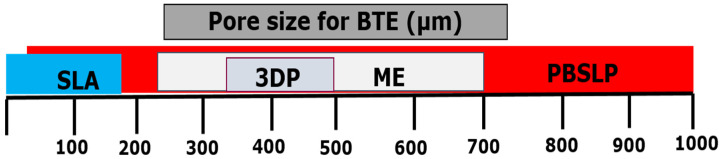
Advantage of PBSLP over other AM techniques used for BTE in terms of porosity of the bioactive scaffolds.

The PBSLP displays several advantages and has the capability of fabricating bioactive porous scaffolds for BTE with pore sizes ranging from 100–1000 µm. Figure 6 depicts the advantage of PBSLP over other AM process in terms of a wide range of the designed porosity needed for bone emulation [47].

### 2.2. Challenges Posed by PBSLP for Bioactive Ceramics

The PBSLP possesses some shortcomings when it comes to powder bed fusion of bioactive ceramic powders. The following shortcomings are focused on this subchapter:

(1) Low production speed for the large-sized scaffolds;
(2) Poor surface quality;
(3) Lack of technical standards, and specific guidelines.

The large-scale product synthesized through PBSLP can distort from the platform when compared to the initial geometry designed by the CAD. The partial and full melting of ceramic particles has several consequences on the 3D porous constructs after laser processing. During partial melting, there are several driving forces acting on the ceramic powders, such as gravity and capillary forces, which can have several repercussions on the consolidation mechanism and surface quality [48]. The gradients in the temperature, known as Marangoni flow, could lead to several crystallographic distortions of the lattice. The thermal stress encountered due to the temperature gradient can lead to a balling effect, unwanted pores, and defect formation [49,50]. The cracking can be minimized by decreasing the layer thickness, so that the staircase effect in the scaffold can be reduced [51]. From a manufacturing point of view, bone mimicking is a challenging procedure as scaffold topologies require particular attention to specific features of the process and materials. For the laser powder-bed processes, sufficient resolution (the laser spot size) depending on the designed architecture, is a tricky task as the thermal cycling that occurs during processing can result in distortion and the ultimate failure of components.

Up to now, the loopholes in the quality assurance systems, manufacturing standard operating procedures and the technical standard for the existing intellectual property rights and regulatory patent law limit the application of customized ceramic parts for BTE [4]. There are challenges to meet the requirements of a specific architectural design for the bioactive scaffold, which are essential to overcome in order to move forward with the development of novel products.

## 3. Bioactive Scaffold Parameters for Bone Tissue Growth

Natural bone tissue is represented by two types of architecture as a cancellous bone possesses up to 90% porosity and a cortical bone has less than 10% porosity. The bioactive ceramic scaffolds for BTE have to meet structural requirements in compliance with the bone’s pore size, porosity level, pore interconnection, and mechanical properties. Scaffolds should be fabricated as biocompatible porous 3D items to ensure mechanical support for cell seeding and a template for tissue regeneration. Herein, the bioactive ceramic structures with the appropriate physical features, bioresorbability, mechanical properties, and biomolecule delivery capabilities which could serve as the ideal candidates to boost bone healing, will be discussed.

### 3.1. Architecture and Porosity

Patient-personalized implants with the required and well-specified properties face geometrical limitations imposed on the scaffolds [52]. The regeneration of bone is supported by the formation of the surrounding tissues. Up to now, the fabrication of supports for different types of cells for osteochondral or vascularized bone regeneration is still challenging [53].

In this regard, suitable porosity may help in increasing the permeability of the body fluids and thus facilitate bone growth [54]. The bioactive porous scaffold facilitates mechanical interlocking with the fractured bone and thus aggregates the functionality at the biointerface [55]. The porous structure can be classified into three categories based on the size of the pores: <100 nm (nanoporous), 100 nm–50 µm (microporous), and >50 µm (macroporous) [56]. The macro- and micropores can be tuned to study the release behavior of various biomolecules, bioactive ions, and other agents onto the scaffold’s structure [57]. Nanopores administer an appropriate microenvironment at the cellular level, which facilitates and assures cell adhesion and promotes a larger amount of protein (laminin, vitronectin, fibronectin, and peptides, etc.), and growth factor attachment [58,59,60]. Porosity is also a rather important parameter in osteoconduction [61]. It has been proven that the fine pore-associated hypoxic conditions can stimulate osteochondral formation before osteogenesis. Large pores can facilitate vascularization resulting in direct osteogenesis [47]. Microporosity can essentially improve the specific surface area of the scaffolds and can further expedite the permeability of fluids into the scaffolds along with accelerating the diffusion of inculcating nutrients and oxygen to the cells by directly communicating with ECM [62,63].

**Figure 7:**
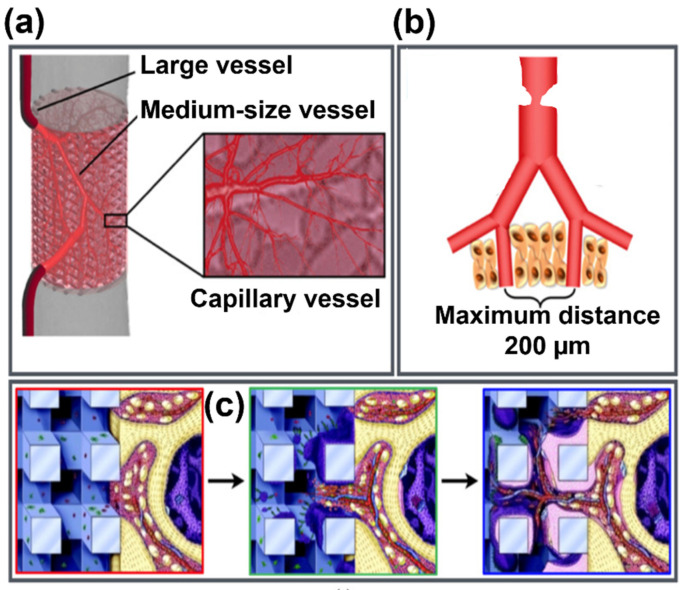
Graphical representation depicting cell migration and vascularization through the pores of the scaffold. (**a**) Vascularization network enhanced by 3D scaffold; (**b**) distance between blood capillaries; (**c**) elucidation of new bone formation around the macropores of the scaffold; (**a**–**c** adapted with permission from [68]).

Macropores with pore sizes in the range of 200–450 µm are the supporting architectural elements needed for new bone tissue growth and vascularization, so that the cells through migration and blood vessels can essentially imbibe both on the intra- and extracellular space (inside and out) of the bioactive scaffold, as illustrated in Figure 7a–c [64]. This can enhance the mineralization process by specifically increasing alkaline phosphatase (ALP) production. Furthermore, the scaffolds should promote cell adhesion, proliferation, and osteoblast migration and, consequently, promote angiogenesis [65,66].

The distribution of porosity is still a debatable question in BTE, as the bone is a complex structure with a variable porosity in different regions depending, in addition, on the physiological state of the patient. Several scaffold architectures with different porosity levels (15–85%) have been studied in the attempt to optimize pore diameters and distribution [47,67,68]. For example, primary rat osteoblasts were seeded onto bioactive scaffolds with differential pore sizes ranging from 40 to 100 µm in [69]. Most of the cells were found clustered around pores of 40 µm followed by the enhanced migration of the cells around pores of 100 µm. β-tricalcium phosphate (β-TCP) scaffolds with a porosity gradient from 80 to 88% with a pore size ranging from 125 to 150 µm subsequently allowed bone growth towards pores of 150 µm [70]. The disparity in the osteogenic study both in vivo and in vitro microenvironments was observed when HA scaffolds with different porosities (70% porosity and 800 μm average pore size versus 60% porosity and 400 μm average pore size) were tested in [71]. It was observed that the goat bone marrow cells (gMSC) proliferated after 6 days in vitro on the scaffolds with 60% porosity; however, on the other hand, the scaffolds implanted into bilateral paraspinal muscles in goats showed a more efficient bone formation on the scaffolds with 70% porosity. A comparative analysis study was made on porous HA scaffolds with a wide variety of pore sizes (106–212, 212–300, 300–400, 400–500, 500–600 μm) [72]. The scaffolds were grafted in rats, and the conclusion that scaffolds with pores size between 300–400 μm demonstrated a higher ALP activity and enhanced osteocalcin (OCN) content with a higher bone growth rate, was drawn by Tsuruga et al. [72].

### 3.2. Mechanical Properties

The mechanical properties (particularly compressive strength and sometimes fatigue) are of special importance and can be customized for a particular purpose by tuning pore architecture and porosity levels. Shao et al. [53] demonstrated several types of CS-bioactive glass composite scaffolds with relatively distinctive uniaxial compressive strengths of 43, 90, and 50 MPa for Archimedean chords, honeycomb, and parallelogram, respectively.

Usually, porous bioactive ceramic scaffolds are considered as a potential replacement for the trabecular or cancellous region of the bone. Only a few works have been reported on the fabrication of single-phase bioactive ceramic scaffolds for long bone or load-bearing defects, which simultaneously promote bone regeneration [73,74,75,76,77].

**Figure 8:**
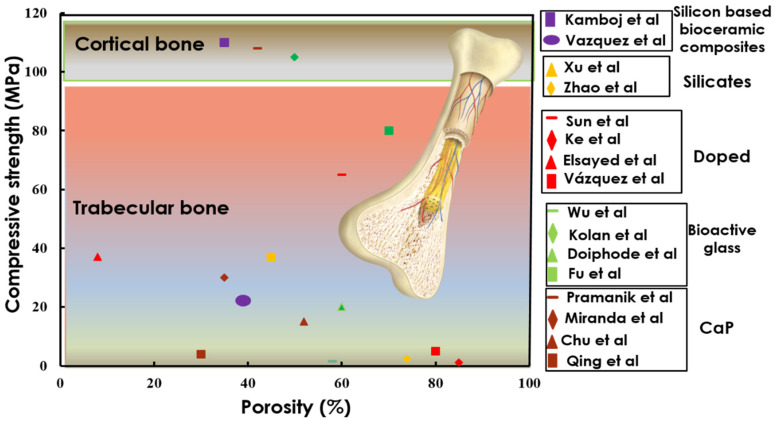
Graphical representation of the bioactive ceramic scaffolds depicting compressive strength vs. porosity, calcium phosphatases [81,82,83,84], bioactive glass ceramics [85,86,87,88], doped bioactive ceramics [89,90,91,92], silicates [93,94], and newly fabricated silicon based bioactive ceramics [17,95]. Adapted with permission from [17].

The bioinert or traditional ceramics usually possess sufficient flexural strength and can be used for load-bearing implants; however, they do not promote angiogenesis and direct bonding to the bone in the same way as bioactive ceramics [78,79,80]. Bioactive ceramics used for critical open bone defect treatments generally experience compressive and traction forces. A wide variety of bioactive ceramic scaffolds, which encompass CaP, doped HA and CS, bioactive glasses and ceramics, and their respective composites, have been used as the constructs for replacing cancellous bone (restoration of the bone loss), shown in Figure 8. Synthetic HA, which is similar to the CaP minerals within bones and teeth, is successfully applied as a bone implant with a suitable mechanical strength and osteoconductivity.

The keyhole pores, apart from the designed porosity from the CAD design, usually affect the mechanical strength of the scaffolds when fabricated through PBSLP. These keyhole pores act as the main concentration point of crack origination in the 3D printed scaffold [96]. However, the mechanical strength is primarily controlled by the pore volume (CAD) of the bioactive scaffolds and usually decreases with the increase in the porosity or pore size, as can be seen in Figure 8.

Furthermore, the compression strength can also be further enhanced by the selection of the bioactive elements. Usually, the CaP and bioactive glass bioceramics usually have lower compression strength when compared to silicon-based bioactive and CS bioceramics. This, particularly, can be attributed to the acicular grains of the CS bioceramics, i.e., the chain structure of the silicates, and belongs to the pyroxenoids, which can be potentially used as fillers to enhance the compression strength of the CaP bioceramics [97]. Among many other properties, fracture toughness should also be carefully considered and designed. Most silicate bioceramics, such as wollastonite (CaSiO_3_), akermanite (Ca_2_Mg[Si_2_O_7_]), diopside (MgCaSi_2_O_6_), and hardystonite (Ca_2_ZnSi_2_O_7_) are generally superior to CaP in terms of fracture resistance [98]. The enhancement in the CS bioceramic fracture resistance can be assigned to the elevation of the destruction viscosity and strength of the scaffold by escalating the work done against the force and ultimately hampering the crack propagation [99]. Although CS bioceramics exhibit relatively higher mechanical properties as compared to CaP, these materials can hardly be used for cortical bone replacement or load-bearing applications. However, on the other hand, silicon-based CS bioactive ceramics fabricated through PBSLP with a large proportion of silicate in the composite can be used for load-bearing applications, also shown in Figure 8 [17].

### 3.3. Biology-Related Characteristics

**Figure 9:**
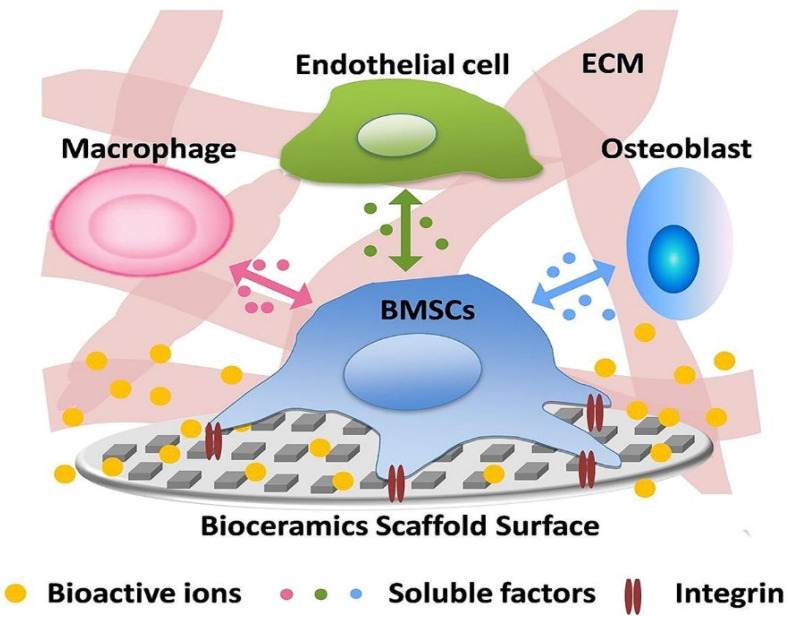
Illustration of the bioactive ceramic scaffold surface with topological and chemical properties, which, as a result, affect the stem cell microenvironment, adapted with permission from [102].

In the case of BTE scaffold, the Kokubo solution has become a common necessity to study the bioactivity and biomineralization process by the formation of biomimetic HA on the surface of novel structures [100]. However, bioactivity and biocompatibility are reflected not only by the formation of HA on the surfaces but also by inducing molecular signaling pathways. The bioactive ceramic scaffolds possess the potential to trigger the signaling molecules, as shown in Figure 9, at the molecular level (involving bioactive ions released from bioceramic scaffold, growth factors, and integrins) without having any adverse effect or cytotoxicity on the host tissue [101].

The bioactivity and biocompatibility ultimately boil down to the bioactive ions released from the surface of the scaffolds. For instance, assorted bioactive ions like Ca, Zn, Si, Mg, and Cu have shown a profound effect in administering the bone regeneration process [52,103]. CaP bioceramics are an important class of biomaterials since the lixiviated Ca^2+^ ions from the scaffold intensify the proliferation of human mesenchymal stem cells (hMSC) due to their bioactivity [104]. The constant delivery of Ca^2+^ ions plays a dynamic role in enhancing the bone regeneration process both in vivo and in vitro [105]. The potential of P^5+^ ions can prompt an osteoinductive response by mineralizing the collagen matrix [106].

Not only can CaP bioceramics affect biocompatibility but CS bioceramics can also enhance and affect the bioactivity and biocompatibility since they can boost the differentiation of hMSC. Silicate-based bioceramics act by stimulating the differentiation of osteomarker genes, such as runt-related transcription factor 2 (Runx2), ALP, middle and later osteogenic markers, such as bone sialoprotein (BSP), OCN, and osteopontin (OPN) [107,108]. The ions released from the silicate-based bioceramics enhance and regulate the expression of the growth factors, such as epidermal growth factor (EGF) and endothelial growth factor (VEGF) of hMSC to facilitate bone regeneration [109,110]. Silicon ions play an essential role in promoting bioactivity in glycosaminoglycan production and nodule formation of the hMSC in the bone regeneration signaling process [111,112,113].

Another class of bioceramic scaffolds derived from bioactive glasses are also promising candidates [114,115]. The ions released from bioglass 45S5 can trigger and activate the vascularization of the endothelial cells resulting in the bone resorption process through the production of the ECM by paracrine signaling [116].

**Figure 10:**
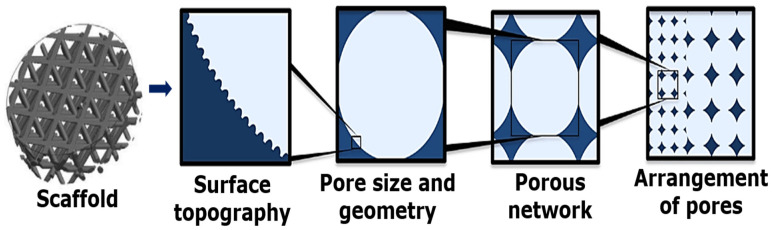
The most influential parameters affecting biodegradation of bioactive scaffold according to the design perspective, adapted from [119].

Biodegradability is another factor to be considered when designing for nature-mimicking scaffolds [117]. The standard degradation rate of the scaffolds should match the new bone ingrowth rate [118]. Figure 10 summarizes the main parameters to be regarded for a successful implementation of the printed bioactive substrate.

Generally, bioactive ceramics fabricated with smaller grains are likely to degrade faster when compared with coarse-grained materials [120]. The nanopores and micropores ensure a large enough contact area and macropores allow the cells to infiltrate to facilitate the degradation process [121,122]. It is also worth noting that topography, surface microstructure (in contact with the body fluids or the site of implantation) in vivo, and mechanical properties usually are bound to change over time during the degradation process and, consequently, affect the stem-cell fate and bone tissue regeneration. However, the scaffold design factors are not solely responsible for the biodegradation process. The choice of the material is of equal importance. The degradation of the CaP bioceramics relies on the solution driven and the stem cell ions (Ca^2+^ and P^5+^) mediated process [123]. However, in contrast, the degradation of the CS-based bioceramics and glasses depends only on the solution mediated or controlled dissolution [120].

All these aforementioned parameters are an essential prerequisite for the fabrication of a bioactive scaffold to be considered as a potential bone graft substitute. PBSLP provides freedom to fine tune the mechanical properties, biodegradability, porosity, and design of the bioactive scaffolds by carefully controlling the exposure of the laser to the bioactive ceramic powder bed.

## 4. Bioactive Ceramic Scaffolds Processed by PBSLP

Bioactive ceramics are of corresponding chemical composition to bone. The commonly used bioactive materials are CaP and silicate-based bioceramics. These materials can stimulate the differentiation of the stem cells to osteoblasts, and are called osteoconductive materials [124]. Bioactive ceramics also have a propensity to directly bond with mangled bone and the potential to form tissue connections in the human body. They are also used to assist critical-sized defects of the bone, based on the size of the fracture.

### 4.1. Bioactive Ceramics

#### 4.1.1. Calcium Phosphate (CaP) Based Ceramics

Herein, two important CaP phases, HA and β-TCP will be detailed, which are processed through PBSLP. HA is well known for its substitution of various ions in its lattice structure [125] and has another edge in terms of advantages concerning bone regeneration since it demonstrates piezoelectric behavior which helps with the acceleration of the bone regeneration process [126]. The scaffolds fabricated with such kinds of materials usually are favored for open bone defects or nonload bearing applications [127].

CaP bioceramics are widely used for bone regeneration processes [128,129], however, the processing of CaP bioceramics through PBSLP is challenging since the impact of the laser on the powder bed may result in excessive grain growth, additional phase development, and decomposition at temperatures of 1250–1300 °C [130]. The additional phases formed can be calcium oxide, α-TCP, and tetra calcium phosphate (TTCP), which may have unpredictable consequences on the mechanical properties of the CaP scaffolds. The potential of a CO_2_ laser operating at 10.6 μm wavelength to sinter the HA powder bed was studied by Shuai et al. [41]. It was demonstrated that varying the energy density from 2 to 4 J/mm^2^ resulted in no other phase formation apart from HA. The scaffold fabricated at 4 J/mm^2^ showed the highest Vickers hardness of 4 GPa and a fracture toughness of 1.28 MPa∙m^1/2^, which is suitable for cancellous bone.

**Figure 11:**
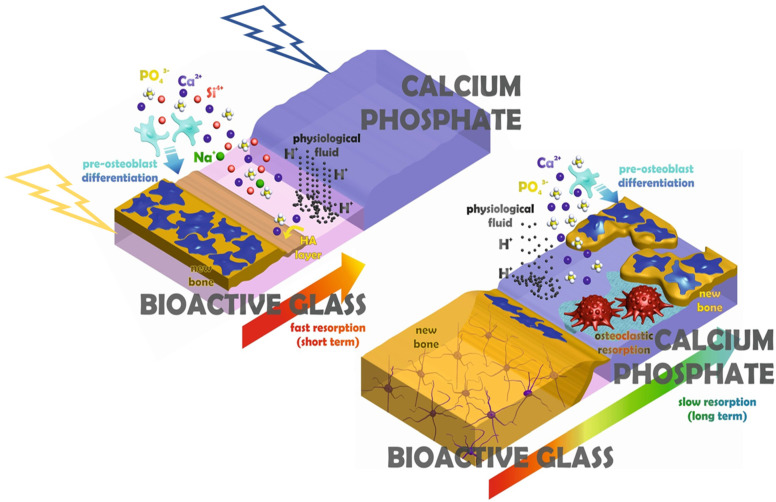
The impact of the different lasers on the fabrication of smart bioactive implants with gradual resorption rates, Nd:YAG laser for CaP component and CO_2_ laser for bioactive-glass component, adapted from [131].

The impact of different lasers can have a profound effect on the performance of the bioactive scaffold by fabricating gradient (smart) implants with different resorption rates, as shown in Figure 11 [131]. The core of the implant constituted a CaP component with the energy density varied between 120 and 150 J/mm^2^ with the impact of Nd:YAG laser, and in comparison, the bioactive glass on the shell was sintered through CO_2_ laser operating at two different wavelengths of 1064 nm and 10,600 nm with the energy density fluctuating from 25 and 45 J/mm^2^. The as-fabricated smart implant incorporating tailored multiphasic CaP on the inner core showed slackened degradation in vitro, which was encircled by sintered bioactive glass particles with a faster degradation rate. The smart bioactive implant fabricated with tailored resorbability under the impact of different lasers can be used for the restoration of craniofacial applications.

Functionally graded CaP bioactive ceramics were studied by Salimi, E [132]. The fabrication of the gradient implant for the osteochondral defect is one of the most important issues among researchers. Therefore, understanding the different phases of the graded CaP bioactive ceramics with the interaction of the laser at the bone−cartilage interphase is considered very important for the tissue-specific interactions [133]. For that matter, the biomechanical functionality aspect co-relating with the assorted phases of the CaP bioactive ceramics is better anticipated for the subchondral bone and calcified cartilage (osteochondral interphase) [134].

Various researchers have focused both on the experimental and numerical study to analyze the behavior of CaP bioceramics under the impact of lasers with different energy densities by changing the scanning speed. The decomposition of the HA was studied with ANSYS software and co-related with the experimental studies in [135]. The decomposition of HA powder with varying energy density on the HA powder bed was attributed to Equations (2)–(4) confirmed by the XRD analysis [42].

Ca_10_(PO_4_)_6_(OH)_2_ = H_2_O + Ca_10_(PO_4_)_6_O (2)

Ca_10_(PO_4_)_6_O = 2Ca_3_(PO_4_)_2_ + Ca_4_P_2_O_9_ (3)

Ca_10_(PO_4_)_6_(OH)_2_ = 2Ca_3_(PO_4_)_2_ + H_2_O + CaO (4)

The initial decomposition temperature of HA with the Ca/P ratio of 1.67 is still debatable. Some authors suggest it is between 1100 °C and 1200 °C [136], and others discovered a temperature of decomposition higher than 1300 °C [137].

Various studies have also implemented metal matrices such as stainless steel, pure titanium, and Ti64 (Ti_6_Al_4_V) along with CaP fillers to improve the osseointegration by direct PBSLP; more specifically SLM of the substrates for load-bearing applications [43,138,139]. However, the metal-HA composite is encountered by the phase transformation of HA due to the higher melting temperature of the metal component as compared to HA. This problem can be overcome by incorporating a polymer matrix along with CaP fillers in the direct PBSLP approach to fabricate polymer-bioceramic bioactive scaffolds [140,141,142,143]. A lower temperature is needed to melt the polymer and, hence, the phase transformation of CaP fillers can be neglected.

The potential of the PBSLP of CaP bioactive ceramics lies in modulating the energy density according to the authors. The varying energy density will escort the formation of the different phases on the CaP powder feedstock to fabricate bioactive smart implants with varying bioactivity and degradability, which can open new avenues for the personalized implants specifically for the osteochondral defects in a single powder feedstock. However, there is still a gap and scope for research to fabricate CaP scaffolds with improved mechanical properties for load-bearing applications, which can essentially be fulfilled by designing triple periodic minimal surface (TPMS) bioactive ceramic scaffolds.

#### 4.1.2. Silicates Based Ceramics

The other class of bioactive materials includes silicate-based bioceramics, which are widely researched and explored and can be considered a substitute for CaP-based bioceramics. The silicate-based bioceramics have shown superior and enhanced apatite-formation ability when compared to the CaP bioceramic counterparts in simulated body conditions [6]. Silicate-based bioceramics encompass CS (calcium silicate, CaSiO_3_), CS-Mg (diopside, akermanite, merwinite, etc.), CS-Zn (hardystonite), CS-Zr (bagdadite), and silicate-based bioglasses. The aforementioned substituted trace elements (Mg, Zn, Zr) in the CS scaffolds have proven to have superior biological and mechanical properties [102].

**Figure 12:**
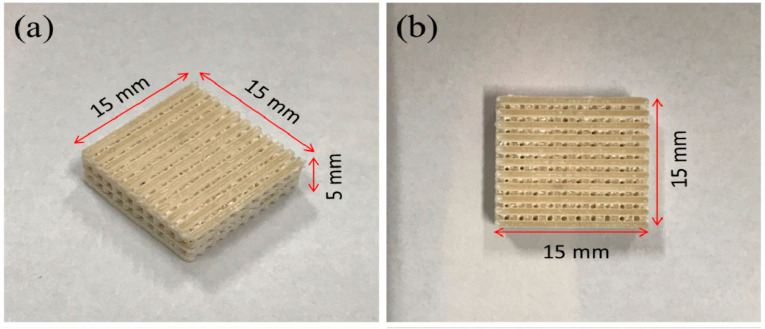
(**a**,**b**) CaSiO_3_-PHBV composite scaffolds with interconnected porosity fabricated through PBSLP method, adapted from [146].

The pure CaSiO_3_ scaffolds have been fabricated through the direct SLS method without involving any binder [144] and have demonstrated that with the increasing laser energy, the low temperature polymorph (β-CaSiO_3_) is transformed to a higher temperature polymorph (α-CaSiO_3_) at 1125 °C. Graphene was also incorporated in the direct powder bed fusion with CaSiO_3_ to form graphene-CaSiO_3_ composite scaffolds with enhanced mechanical properties of the bioactive scaffolds [145]. In this study too, the conversion of β-CaSiO_3_ to α-CaSiO_3_ with the impact of increasing laser energy is observed_._ Furthermore, the bioactivity of CS bioceramic was enhanced by incorporating Poly(3-hydroxybutyrate-*co*-3-hydroxyvalerate) (PHBV) into the powder bed by direct SLS process. The PHBV/CS composite scaffolds proved to accelerate the proliferation and osteogenic differentiation of stem cells by mimicking Collagen Type 1 (COL-1) when compared to the pure CaSiO_3_ scaffolds, as shown in Figure 12 [146].

Diopside-based composite scaffolds reinforced with graphene nanoplatelets (GNP) with a few layers of graphene were also studied through PBSLP. The composite scaffolds exhibited enhanced bioactivity, compression strength, and fracture toughness (toughening mechanism of GNP) when studied against pure diopside scaffolds [147]. Similarly, akermanite-reinforced PHBV composite scaffolds were also studied [148]. They comprehensively studied the effect of akermanite particles (micro/nano) affecting mechanical properties, water up-taking ability on the composite akermanite/PHBV scaffolds.

However, the fabrication of the silicate-based bioceramics and glasses are very limited to the Nd:YAG when compared to the CO_2_ laser. This can be attributed to the lower absorbance of silicate-based bioceramics under the impact of the Nd:YAG laser [149]. The prospects of fabricating novel silicate-based bioceramics and silicate-based bioglass scaffolds (designed by the phase diagram of CaSiO_3_–Ca_3_(PO_4_)_2_–MgCa(SiO_3_)_2_) by incorporating silicon in the powder bed through Nd:YAG laser are shown in [17,95,150]. The potential of pore-driven osteogenesis and sustained delivery of antibiotic vancomycin through silicon-based bioceramic scaffolds was also disclosed [35,150]. Silicon has already displayed its potential in biocalcification, biomineralization, osteogenesis, and BTE [151,152,153]. The potential of pure silicon in the powder bed through Nd:YAG laser interaction was also unveiled [154]. Apart from the earthbound silica-based biomaterial applications in the biomedical sector, silica-based material research through SLM has been further extended to other extraterrestrial planets like the Moon and Mars because of their abundance [155].

## 5. Synthetic Bioactive Ceramic Scaffolds for Critical Size Bone Defects by PBSLP

**Figure 13:**
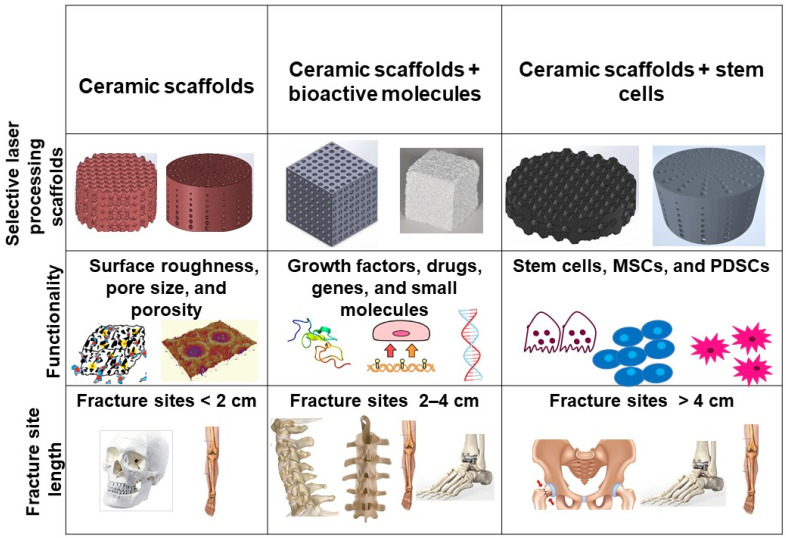
Three major categories of synthetic bioactive ceramic scaffolds processed by PBSLP used for bone repair.

Bone defects can be characterized into different classifications according to the position of the fracture: long bones, assorted regions of the spinal cord, maxillofacial and craniofacial. The most frequent bone fracture sites are the femur, shoulder, hip, wrist (radius, ulna), tibia, ankle (distal tibia/fibula fractures), vertebral, maxilla-, and craniofacial (jawbone, calvaria) fractures [156]. The fractures also depend on the size and the length at the damage sites, as shown in Figure 13.

Critical-sized bone defects usually range between 1 and 4.5 cm [157]. A permanent, direct, and sustainable solution is a requisite to overcome the imperfections of natural bone scaffolds to heal and repair critical-sized bone defects. Synthetic bioactive ceramic scaffolds mask an indispensable role in BTE, which therefore can mimic the natural bone characteristics such as providing support similar to ECM by facilitating and providing a 3D network for regenerating the defected bone. The critical-sized defects of bone can be refurbished by bioactive scaffolds through the assorted added functionalities of 3D printing, such as surface roughness, pore design, porosity, and infusion of cell-based targeted functionality through the scaffold. Scaffolds are engineered to heal bone depending on the severity of the trauma. The three main divisions or strategies for bone repair using ceramic elements involving PBSLP in Figure 13 encompass: (i) 3D synthetic bioactive scaffolds, (ii) synthetic bioactive scaffolds combined with active moieties; and (iii) synthetic bioactive scaffolds in combination with cell-based products.

Nowadays, 3D printing has emanated as a competent solution for the fabrication of scaffolds for BTE, as its scaffold designs perfectly match with the defective site. Diversifying additive manufacturing techniques allow superior control of the parameters for the fabrication of synthetic scaffolds; however, PBSLP similar to SLM/SLS allows a wide range of control over pore size, porosity, and interconnectivity of the scaffolds [158] to better suit the anatomy of the patient’s defective bone, also described in detail in Section 2.2. The main advantage associated with the synthetic bioactive ceramic bone grafts fabricated through SLM/SLS is the ability to fine-tune the mechanical properties and administer degradation rate. Additionally, the bioactive ceramic-based scaffolds fabricated by SLM possess the potential to be used for both cortical and trabecular bone applications [17,150].

In BTE, osteoconductive synthetic bioactive ceramic scaffolds alone sometimes cannot cause rapid healing and rejuvenation of the lost bone tissue due to the paucity of active biomolecules, which cannot promote cell differentiation and proliferation. Growth factors, genes, and stem cell therapy are vital quintessence for instantaneous bone healing, morphogenesis, and tissue rejuvenation since they are osteoinductive. Bioactive scaffolds manufactured by PBSLP can also be integrated with active biomolecules to achieve active biomolecule delivery with the required loading and efficacy to the defective bone (clinical target), as shown in column II and III of Figure 13.

The stability of the implant is also of prime importance when processed through PBSLP, as mentioned in Figure 13, which is primarily dependent on fracture site length. Defects, mostly in the cranium, with a size < 2 cm are predominately healed through direct or intramembranous ossification [159], which is generally regarded as a mechanically stable defect when compared with column II and III of Figure 13. The microsurface roughness (1 µm–0.1 mm) imparted to the scaffolds by the PBSLP process [20] promotes firm anchorage to the fractured bone and formation of the new bone tissue in and around the scaffold [160]. The critical fracture defects usually of >2 cm as depicted in column II and III of the Figure 13, mostly prevailing in the long bones, are generally repaired through endochondral ossification by forming cartilaginous callus, and are regarded as mechanically unstable fractures due to their large size [156]. The biofunctionalized active moieties, such as stem cells and growth factors, on the ceramic scaffolds processed through PBSLP could initiate the immediate formation of an avascular callus of cartilage. The bioactive moieties facilitate the endochondral process [161], while the porous ceramic scaffold processed through PBSLP provides the necessary mechanical stability needed for the bone defect (load-bearing applications).

**Figure 14:**
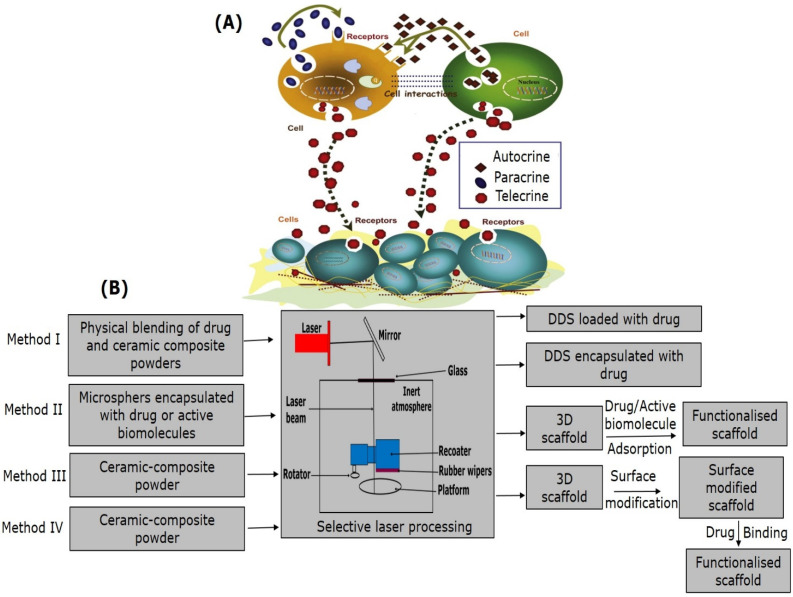
(**A**) Illustration of the mode of the activity of the growth factor by autocrine, paracrine and telecrine signaling pathways, adapted with permission from [163], (**B**) Assorted strategies for developing DDS system using PBSLP.

There are several methods through which growth factors result in tissue repair and formation since they are associated with cell proliferation and differentiation, therefore leading to scar formation or bone tissue repair [162]. Growth factor delivery systems and the mechanism of the action on the various cells can be broadly classified into three categories as explained in Figure 14A, which can be autocrine (act on the same cells through which they are secreted), paracrine (act on the adjacent cells or those in the vicinity), and telecrine (act on distant cells) signaling [163]. PBSLP can act as a meditator for the incorporation of the growth factors and/or drugs. Four different methods have been proposed for the development of DDS by PBSLP, as mentioned in Figure 14B [9]. These methods can be broadly classified into two broad strategies. The first strategy (Method I, II, in Figure 14B) includes the blending of the active biomolecules along with the bioactive ceramic powders in the powder bed. The second strategy comprises firstly, the processing of the bioactive ceramic powders by SLM/SLS and secondly, functionalizing the scaffold through the active biomolecules (Method III, IV, in Figure 14B). However, the heat generated in the PBSLP can bring about damage to the biomolecules incorporated in the powder bed system and can degrade them as followed in Method (I, II) [164]. In order to bypass this approach, the easiest way would be to incorporate active biomolecules after the fabrication of the synthetic bioactive ceramic scaffolds through surface functionalization [165,166]. This approach would provide a customized release of the active biomolecules since scaffolds fabricated through PBSLP can be tailored based on the pore size, porosity, surface roughness, and surface modification desired for BTE. Recently, Kulinowski et al. [10] showed the potential of the SLS technology as an efficient way to tune the release of the active biomolecules by changing the hatch space parameter. Implants were fabricated for drug delivery through SLS technology with contorted shapes such as gyroids for BTE [33]. It was also shown that the potential of new formulations of silicon-CS-polymer composite constructs by SLM technology can be used for the delivery of antibiotic (vancomycin) to eradicate the infection caused by *Staphylococci aureus* and can lead to chronic osteomyelitis [35]. These orthobiologic materials or bioactive ceramic synthetic grafts constructed by SLM/SLS also act as mediators for the delivery of the drugs, growth factors, and stem cells.

The commonly used growth factors for BTE incorporate angiogenic and osteogenic insulin-like growth factor (IGF), bone morphogenetic protein (BMPs), platelet-derived growth factor (PDGF), transforming growth factor beta (TGFβ), and inflammatory factors like interleukins, and macrophage colony-stimulating factor. As an efficient way to deliver the osteoinductive growth factors to the delivery site, they should reach the target site without any loss of bioactivity to achieve the maximum therapeutic effect [163]. The sustained release of growth factor recombinant human BMPs (rhBMP-2) was demonstrated through bioactive CaP/PHBV scaffolds fabricated through SLS with the surface modified through gelatin by physical entrapment [167]. It was also demonstrated through the micro-CT data that the scaffold without rhBMP-2 growth factor showed very limited bone formation around the CaP/PHBV scaffold when compared to the one loaded with growth factor. These growth factors and/or active biomolecules augment the bone regeneration or bone hemostasis by three different distinct steps as suggested in [168], which include (a) inflammation at the damaged site of the bone, (b) soft callus formation at the damaged site through which vascularization of the newly formed bone occurs (angiogenesis), and (c) differentiation of the mobilized stem cells to osteoblasts at the damaged site by hard callus formation.

Having said that the stem cells are recruited to the damaged site of the bone for hard callus formation, the osteogenic progenitor cells naturally secrete growth factors, which therefore can be directly seeded onto the scaffolds to speed up the recovery at the damaged site along with the surplus amount of growth factor supply at the damaged bone for enhanced vascularization. Various researchers have tried to seed stem cells onto the bioactive ceramic and/or composite scaffolds fabricated through PBSLP. The stem cells immobilized onto the ceramic coupons generally are platelet-derived stem cells (PDSCs), bone marrow-derived stem cells (BMSCs), and adipose-derived stem cells (ASCs).

The fabrication of [169] customized apatite-wollastonite glass-ceramic scaffolds by SLS process by recruiting stem cells taken from the femoral head has been reported for its osteosupportive capacity. The scaffolds comprising the polymer and bioactive ceramic component can further enhance the osteogenesis since scaffolds mimic both the ceramic and organic components of the bone. The ASCs were incorporated [170] onto the polycarpolactone-TCP-collagen scaffolds with interporous connections by the SLS process. These bioactive composite scaffolds have been reported to enhance the proliferation and osteogenic differentiation of ASCs cells, both in vitro and in vivo environments. Additionally, cells seeded onto the scaffolds (platform) can be genetically engineered ex vivo for the continuous and/or increased secretion of the growth factors by a variety of DDS [171,172]. Thus, the unification of bone tissue engineering and synthetic bioactive ceramic bone scaffolds using PBSLP has become a synergistic approach, nowadays, for repairing critical-sized bone defects which depend on the loading of active biomolecules. Furthermore, 3D-customized bioactive ceramic scaffolds are a current trend, as they are biocompatible, osteoconductive, and can precisely fit into a defect in a patient’s cranial, maxillofacial, femur, and tibia bones for critical-sized defects.

## 6. Conclusions Remarks and Potential Developments

AM-produced structures have significantly progressed in tissue engineering, providing a basis for customized design and tunable properties. In terms of the processing of metallic and polymeric structures, and their composites, the methods and approaches are fully developed by now. This review focuses on the fabrication of bioactive ceramic scaffolds, mostly on CaP and CS through direct PBSLP. The direct PBSLP displays much potential in the manufacturing of bioactive ceramic scaffolds for BTE and undoubted advantages over other AM techniques due to the capability to tailor the porosity over a wide range.

The requirements of the bioactive ceramic scaffolds for BTE are presented in accordance with the architecture, porosity, bioactivity, biodegradability, and mechanical properties. Usually, bioactive ceramic powders are not susceptible to absorbing laser beams at a lower wavelength with higher frequency (Nd:YAG, Yb:YAG). One potential solution can be the mixing of the additive along with the ceramic powder bed. The additive itself should have a good absorption coefficient, biocompatibility, and osteoconductivity.

PBSLP has the potential to fabricate scaffolds with incorporated drugs and biomolecules straightaway into the powder bed or they can be functionalized with the active moieties after the fabrication. Different classifications of DDS have also been drawn by unlocking the potential of PBSLP. The customization or programming of the drugs release can be tailored by tuning the process parameters, such as hatch distance and scanning speed. The customized biomimetic constructs produced by PBSLP could be a solution for personalized medicine for bone repair in the near future by targeting specific patient groups.

Still, there are challenges in the design and fabrication of an “ideal” scaffold, which can fit both the dense and the porous part of the bone. One of the solutions is the development of a triple periodic minimal surface (TPMS) through an architectural design that would allow mechanical loads inserted into the structure to be withstood and the permeability of body fluids to be significantly increased. Metal porous TPMSs can be easily fabricated and are well known for BTE [173,174,175,176,177,178,179]. However, on the other hand, there is very limited progress and development of TPMS bioactive ceramic surfaces, although there are some recent studies in the literature [76,180,181]. More and more efforts are currently dedicated to topology optimization and fundamental material design. The demand for customized scaffolds has forced the research on bioactive ceramics in terms of process properties optimization and printing both patient-specific and market-attractive constructs.

## Data Availability

Not applicable.
